# Generation of novel microcarrier for expansion of human mesenchymal stem cells

**DOI:** 10.1186/1753-6561-9-S9-P71

**Published:** 2015-12-14

**Authors:** Dave Splan, Grishma Patel, Heather Woolls, Mark Szczypka

**Affiliations:** 1 Pall Life Sciences, 4370 Varsity Drive, Ann Arbor Michigan 48108, USA

## Background

Multipotent stem cells have been isolated from multiple sources including, bone marrow, adipose tissue, placenta, umbilical cord and cardiac tissue. It is predicted that large numbers of therapeutically-active cells isolated from these tissue sources will be required to treat patients inflicted with various disorders. Experimental evidence suggests that these various cell types can exhibit distinct characteristics depending upon tissue source and method of expansion: differential expression of cellular markers is sometimes detected, doubling times and expansion limits can differ, and physical differences that influence the ability of cells to adhere to various synthetic surfaces are observed. A novel prototype microcarrier recently developed by Pall promotes rapid attachment and growth of multiple cell types in stirred-tank reactors. Additionally, peptide-coating provides an alternative animal component-free substrate for cell expansion. These desirable attributes manifest in both serum-containing and animal component-free medium formulations.

## Materials and methods

**Figure 1:**
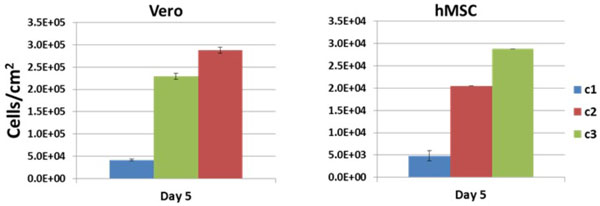
**Microcarrier surface peptide concentrations were varied and cell attachment and growth was assessed**. Microcarriers were coated with recombinant peptide at various concentrations (C1 to C3). Visual observation of attachment kinetics indicates that the concentration of peptide used to coat microcarriers influenced the attachment efficiency as greater numbers of unattached cells were observed at the C1 coating concentration when compared to C2 and C3. Optimal attachment was achieved at C3 coating concentration as observed for both Vero and hMSCs.

A spherical microcarrier with a chemistry that promotes rapid attachment and superior growth of multiple cell types under a variety of environmental condition has recently been developed. The microcarrier chemistry was optimized through an iterative process using these parameters to guide development. Figure 1 shows the cell growth responses to a range of surface chemistry concentrations. Cell attachment and growth in medium containing high concentrations of serum was quantified, and microcarriers that provided the best substrate for attachment and growth were selected for further analysis. Once an optimal surface chemistry was achieved, the ability to expand human bone marrow-derived mesenchymal stem cells in stirred reactors was tested. Initial growth and optimization studies were performed in small scale spinners. Cell densities, doubling times and maintenance of identity and function were ascertained after culture in stirred tank culture. Favorable conditions identified at small scale were then examined in environmentally-controlled bioreactors. Cell harvest efficiencies at small scale and bioreactor levels were optimized.

## Results

Human bone marrow-derived mesenchymal stem cells expanded on this new microcarrier type reached acceptable cell densities in spinner cultures under a variety of environmental conditions. Harvest efficiencies achieved in small scale cultures were excellent, and cell identity was maintained. Conditions optimized in small-scale spinners were successfully employed in environmentally-controlled bioreactor. Cell harvesting optimization studies at larger scale are currently underway. Results to-date indicate that this novel microcarrier type will provide a superior substrate for large-scale propagation of MSCs under various environmental conditions.

## Conclusions

In conclusion, two novel animal protein-free microcarrier types that support excellent attachment and growth of human mesenchymal stem cells were generated; optimal surface charge density which promotes rapid cell attachment and subsequent growth was identified; peptide concentrations and coating conditions that support efficient growth of hMSCs were identified; excellent growth of cells was achieved; cell harvest at small scale demonstrated efficient removal of cells with standard enzymatic treatment; cell growth on novel microcarrier types was similar to that achieved with commercially-available collagen-coated microcarriers; environmental conditions were optimized to support excellent growth and harvest efficiencies from two liter bioreactors yielded reached 1 to 2.5 billion cells. Excellent harvest efficiencies from microcarriers were obtained using enzymatic treatment and application of moderate shear force. Harvest efficiencies of 97% with >95% viability were obtained with hMSC. Human mesenchymal stem cells retained the ability to differentiate into adipocytes and osteocytes after growth on novel microcarrier types.

